# MRI and Prenatal Alcohol Exposure: Images Provide Insight Into FAS

**Published:** 1994

**Authors:** Sarah N. Mattson, Terry L. Jernigan, Edward P. Riley

**Affiliations:** Sarah N. Mattson, M.A., is a graduate student, and Edward P. Riley, Ph.D., is a professor in the San Diego State University/ University of California, San Diego Joint Doctoral Program in Clinical Psychology in the Department of Psychology, San Diego State University, San Diego, California. Terry L. Jernigan, Ph.D., is a staff psychologist in the Psychology Service, VA Medical Center, San Diego, and associate professor in the Departments of Psychiatry and Radiology, University of California, San Diego, School of Medicine, San Diego, California

## Abstract

Magnetic resonance imaging reveals brain abnormalities in some children exposed prenatally to alcohol. Researchers have begun to associate specific abnormalities with features such as memory deficits that are exhibited by these children.

Twenty years have passed since the damaging effects of heavy prenatal alcohol exposure^1^ on children were first noted in the United States (Jones et al. 1973). A consistent pattern of facial malformations, growth retardation, and varying degrees of central nervous system dysfunction became known as fetal alcohol syndrome (FAS). During an autopsy, the brain of an infant who died at 5 days of age was found to be extremely abnormal (Jones and Smith 1975). The infant, whose condition was one of the first to be diagnosed as FAS, had a small brain that was missing the corpus callosum (see figure 1 for a description and diagram of this and other brain areas), a structure that allows information to be transmitted between the two brain hemispheres. During normal embryological development, the nerve cells and the cells that give them structural support migrate to their appropriate positions in the brain. In this infant, the autopsy showed that throughout the brain tissue, there were cells, which could be differentiated by appearance, that had settled in inappropriate places.

Other reports of brain anomalies in children with FAS include increased fluid in the brain, or hydrocephalus; abnormalities of specific areas of the brain, such as the corpus callosum and cerebellum; and evidence of disorganized cell migration during development (Clarren 1986). Some of these findings have been supported by research that uses animal models of prenatal alcohol exposure. Rats and mice exposed to alcohol before birth have been shown to have numerous abnormalities in the brain, including an abnormally small head (microcephaly); altered migration of brain cells, or neurons; and abnormalities of the cerebellum (Miller 1993; West and Pierce 1986). The cerebellum is the structure responsible for coordination of movement and posture and is also believed to have a role in some aspects of cognition.

## Magnetic Resonance Imaging

Recently, new technology has allowed researchers to use a noninvasive technique to look at brains of living children. Magnetic resonance imaging (MRI) produces x-raylike pictures of the brain without using radiation. MRI works like this: a large magnet causes the atoms in the brain to line up in a consistent fashion. An electromagnetic signal then passes through the magnetic field and provides energy that is absorbed by the aligned atoms. When the electromagnetic signal is discontinued, the atoms release measurable amounts of energy. Different tissues in the brain, such as gray matter and white matter, that have different chemical compositions absorb, and thus release, energy in different ways. For example, the corpus callosum, which is primarily white matter, is distinguishable on an MRI from the cerebellum, which is mainly gray matter. These energy signals are converted to pictures of the brain (Jernigan 1990). Magnetic resonance images can appear as three-dimensional pictures or can be divided into two-dimensional “slices” of specific areas of interest. This article addresses the use of MRI in ongoing research on brain structure and function in FAS.

## Research Findings

In continuing research, we have conducted MRI evaluations on 12 children whose mothers were known to consume large amounts of alcohol while pregnant; 4 of the children have been subjected to detailed quantitative evaluations. In these four children, the volumes of particular brain areas were determined for comparison with the volumes of these areas in normal, healthy children. Two of these cases were adolescents with a clinical diagnosis of FAS (Mattson et al. 1992). The other two cases were adolescents with a known history of heavy in utero alcohol exposure but who lacked sufficient characteristics to warrant the clinical diagnosis of FAS (Mattson et al. in press *a*). These two cases are referred to as “PEA,” for prenatal exposure to alcohol.

MRI evaluations revealed that all four children were microcephalic. The volume of each adolescent’s brain was, on average, about 25 percent smaller than the brain of a healthy child of the same age. Similarly, the volume of the cerebellar area was smaller than that of a healthy child by approximately 20 percent.

## Effects on Basal Ganglia

One group of structures studied in the four children that appeared to be particularly affected was the basal ganglia. The basal ganglia lie deep in the brain and are involved in both movement and cognition; there are extensive nerve fiber connections between the basal ganglia and the outer portion of the brain, or cortex.

We controlled for the overall reduction in the volume of the brain in the children by looking at the volume of each brain part in proportion to its overall volume. Even with this method of control, the basal ganglia were still reduced in size, compared with those of both normal and mentally retarded control subjects. There was little difference in the volumes of the overall brain and the basal ganglia between the two children with FAS and the two with PEA.

The volume of the diencephalon (an area of the brain that encompasses the thalamus, which is the brain’s relay center; the hypothalamus, which is responsible for regulating the pituitary gland; and the septal area, which is related to the limbic system) was reduced in all four children. However, when we controlled for the overall reduction in brain size, an interesting difference emerged between the children with FAS and those with PEA. The two children with FAS showed a reduction in the proportional size of the diencephalon, whereas even though the children with PEA showed a reduction, the ratio remained within the normal range for healthy control subjects. Thus, the diencephalon may only be reduced in proportional volume in severe cases of alcohol exposure (e.g., FAS), whereas the proportional size of the basal ganglia compared with total brain volume may be reduced following heavy gestational alcohol exposure, both for PEA and for FAS subjects.

## Effects on the Corpus Callosum

**Figure 2:**
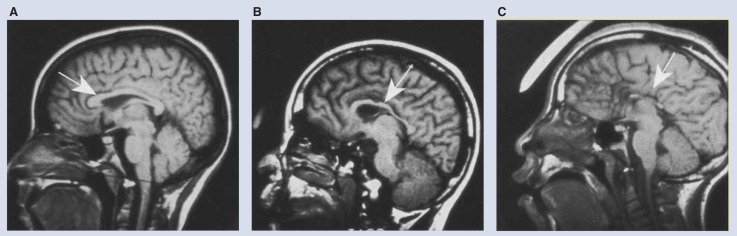
(A) MRI showing the side view of a 14-year-old control subject with a normal corpus callosum, (B) a 12-year-old with FAS and a thin corpus callosum, and (C) a 14-year-old with FAS and agenesis (i.e., absence due to abnormal development) of the corpus callosum. MRI’s courtesy of the authors.

As mentioned above, the corpus callosum connects the hemispheres of the brain and allows information to be transmitted between them. In general, the right hemisphere of the brain controls the left side of the body, and the left hemisphere of the brain controls the right side of the body. The corpus callosum allows both hemispheres to know what happens in both sides of the body. The first child with FAS that we assessed using MRI, who was one of the two FAS children evaluated in the basal ganglia and diencephalon research, did not have a corpus callosum. The corpus callosum does not appear to be essential for normal functioning, but its absence is a relatively rare abnormality that occurs in about 0.1 percent of the general population. In the learning disabled and mentally retarded population, absence of the corpus callosum is more common, occurring in about 2 percent of that population (Jeret et al. 1986). Two other children in our sample lack the corpus callosum. Figure 2 shows an MRI scan of a child with FAS who lacks a corpus callosum as well as a scan of a child with FAS who shows thinning in the back part of the corpus callosum.

The corpus callosum also was investigated in 10 children whose mothers were heavy drinkers during their pregnancies. The overall area of the corpus callosum, taken using two-dimensional slices of magnetic resonance images, is smaller in children in the alcohol-exposed group than in a control group of normal children who were matched to them for age and gender. In addition, when we divided the corpus callosum into regions, a standard method for analyzing this structure, four out of the five regions were significantly smaller in the alcohol-exposed children than the same regions in the normal control group children.

Because the alcohol-exposed children have smaller brains overall, we also looked at their corpora callosa in proportion to their overall brain sizes, as we had done for the basal ganglia and diencephalon. Even in this assessment, the alcohol-exposed group still showed three corpus callosum regions that were significantly smaller than those in the normal control group. At this time, we do not fully understand the causes, nor can we predict the consequences, of this size difference. It may be that in the three proportionally reduced regions, there are fewer axons, or nerve fibers, crossing between the hemispheres, or it is possible that there are a normal number of extraordinarily small-diameter axons crossing. In either case, one consequence of a reduced corpus callosum might be abnormal development of the division of responsibilities that generally takes place between the hemispheres.

## Association of MRI Findings With Prenatal Alcohol Exposure

The information gained from MRI should help researchers make connections between the behavioral and cognitive characteristics, such as memory deficits, that are associated with heavy prenatal alcohol exposure and specific structural defects in the brain.

One example that relates brain structure to function in adults is Huntington’s disease (HD). It is an inherited disorder associated with damage to the basal ganglia that causes motor abnormalities as well as memory problems, or dementia. The problems with memory include difficulty in retrieving stored information. For example, on the California Verbal Learning Test (CVLT), a test that measures verbal learning and memory, patients with HD show limited ability in learning and immediately recalling a word list. Specifically, they display repetitive (perseverative) errors; are insensitive to proactive interference (which is when old information interferes with the learning of new information); and perform better when asked, in a yes/no format, whether certain words appeared on the list.

The CVLT for children has been given to FAS children, and the results suggest a pattern of performance somewhat similar to that of the HD patients (Mattson et al. 1991). Because HD patients’ disabilities are associated with basal ganglia damage, and MRI findings show FAS children to have proportionally reduced basal ganglia, the similarities between the two disorders suggest the possibility that the smaller basal ganglia size may affect FAS children’s memory problems.

The basal ganglia are known to play a role in a person’s ability to remember where things are in space, in a person’s goal-directed behavior, and in a person’s ability to successfully transfer from one activity or task to the next (Cote and Crutcher 1991). Children with FAS have been reported to have deficits in memory, specifically spatial memory; to have difficulty learning the consequences of their actions; and to be perseverative. These behaviors may be related to the small volume of the basal ganglia that is seen in children with histories of heavy prenatal alcohol exposure, not just in those with FAS (Mattson et al. in press). Other brain areas also may be involved in the behavioral effects that result from alcohol exposure. For example, the diencephalon is reduced in FAS children (although it is not in PEA children), and this may contribute to their generally poorer behavioral functioning, compared with the PEA children.

The findings regarding the corpus callosum are interesting as well. The three regions of the corpus callosum that are reduced in prenatally alcohol-exposed children are, in healthy people, thought to contain nerve fibers from several parts of the brain, including the frontal region, involved in complex integration of other brain systems; the posterior parietal region, involved in visual and spatial functioning; the temporal region, involved in memory; and the occipital region, involved in vision. Interestingly, these corpus callosum regions also are reduced in children with attention-deficit hyperactivity disorder (ADHD) (Hynd et al. 1991). Deficits in attention, such as becoming easily distracted from a task, and increased activity have long been considered signs of FAS (Streissguth 1986). In addition, the specific brain regions that are reduced in children in both the ADHD and the FAS groups may reflect some common underlying dysfunction in brain development that appears in children with either malady. For example, children with FAS or ADHD may have underlying deficiencies related to the frontal regions of the brain, as evidenced by their motor persistence, such as hyperactivity, and their difficulty inhibiting unwanted behaviors.

## Conclusions

MRI allows researchers to view the brains of living children who have been exposed to large amounts of alcohol in the womb. Ultimately, the patterns of malformations seen in the brain can be related to the behavioral profiles of children with FAS and other alcohol-related birth defects. Correlating brain structure to function may make it easier to understand the mechanisms behind the potentially devastating effects that alcohol has on the developing fetus.

## Figures and Tables

**Figure 1:**
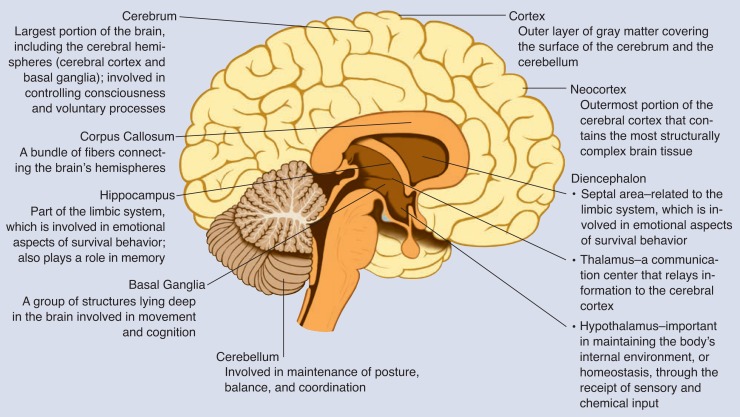
Areas of the brain that can be damaged in utero by maternal alcohol consumption.

## Footnotes

1 Mothers of children mentioned in this article are known to have consumed substantial amounts of alcohol during their pregnancies. However, terms such as “heavy prenatal exposure” and “heavy drinkers,” as they are used in the article, do not refer to a specific amount of alcohol. It is not known exactly how much the women drank during their pregnancies.

## References

[b1-arhw-18-1-49] Clarren SK, West JR (1986). Neuropathology in fetal alcohol syndrome. Alcohol and Brain Development.

[b2-arhw-18-1-49] Cote L, Crutcher MD, Kandel ER, Schwartz JH, Jessell TM (1991). The basal ganglia. Principles of Neural Science.

[b3-arhw-18-1-49] Hynd GW, Semrud-Clikeman M, Lorys AR, Novey ES, Eliopulos D, Lyytinen H (1991). Corpus callosum morphology in attention-deficit hyperactivity disorder: Morphometric analysis of MRI. Journal of Learning Disorders.

[b4-arhw-18-1-49] Jeret JS, Serur D, Wisniewski K, Fisch C (1986). Frequency of agenesis of the corpus callosum in the developmentally disabled population as determined by computerized tomography. Pediatric Neurosciences.

[b5-arhw-18-1-49] Jernigan TL, Baker GB, Hiscock M (1990). Techniques for imaging brain structure: Neuropsychological applications. Neuromethods: Volume 17. Neuropsychology.

[b6-arhw-18-1-49] Jones KL, Smith DW (1975). The fetal alcohol syndrome. Teratology.

[b7-arhw-18-1-49] Jones KL, Smith DW, Ulleland CW, Streissguth AP (1973). Pattern of malformation in offspring of chronic alcoholic women. Lancet.

[b8-arhw-18-1-49] Mattson SN, Stern C, Jones KL, Delis DC, Riley EP (1991). Verbal learning and memory in children with fetal alcohol syndrome (Abstract). Alcoholism: Clinical and Experimental Research.

[b9-arhw-18-1-49] Mattson SN, Riley EP, Jernigan TL, Ehlers CL, Delis DC, Jones KL, Stern C, Johnson KA, Hesselink JR, Bellugi U (1992). Fetal alcohol syndrome: A case report of neuropsychological, MRI, and EEG assessment of two children. Alcoholism: Clinical and Experimental Research.

[b10-arhw-18-1-49] Mattson SN, Riley EP, Jernigan TL, Garcia A, Kaneko WM, Ehlers CL, Jones KL A decrease in the size of the basal ganglia following prenatal alcohol exposure. Neurotoxicology and Teratology.

[b11-arhw-18-1-49] Miller MW (1993). Migration of cortical neurons is altered by gestational exposure to ethanol. Alcoholism: Clinical and Experimental Research.

[b12-arhw-18-1-49] Streissguth AP, West JR (1986). The behavioral teratology of alcohol: Performance, behavioral, and intellectual deficits in prenatally exposed children. Alcohol and Brain Development.

[b13-arhw-18-1-49] West JR, Pierce DR, West JR (1986). Perinatal alcohol exposure and neuronal damage. Alcohol and Brain Development.

